# Alpha-tocopherol in intravenous lipid emulsions imparts hepatic protection in a murine model of hepatosteatosis induced by the enteral administration of a parenteral nutrition solution

**DOI:** 10.1371/journal.pone.0217155

**Published:** 2019-07-11

**Authors:** Gillian L. Fell, Lorenzo Anez-Bustillos, Duy T. Dao, Meredith A. Baker, Prathima Nandivada, Bennet S. Cho, Amy Pan, Alison A. O’Loughlin, Vania Nose, Kathleen M. Gura, Mark Puder

**Affiliations:** 1 Vascular Biology Program, Department of Surgery, Boston Children’s Hospital, Boston, Massachusetts, United States of America; 2 Department of Surgery, Beth Israel Deaconess Medical Center, Boston, Massachusetts, United States of America; 3 Department of Pathology, Massachusetts General Hospital, Boston, Massachusetts, United States of America; 4 Department of Pharmacy, Boston Children’s Hospital, Boston, Massachusetts, United States of America; Max Delbrueck Center for Molecular Medicine, GERMANY

## Abstract

Intestinal failure-associated liver disease (IFALD) is a risk of parenteral nutrition (PN)-dependence. Intravenous soybean oil-based parenteral fat can exacerbate the risk of IFALD while intravenous fish oil can minimize its progression, yet the mechanisms by which soybean oil harms and fish oil protects the liver are uncertain. Properties that differentiate soybean and fish oils include α-tocopherol and phytosterol content. Soybean oil is rich in phytosterols and contains little α-tocopherol. Fish oil contains abundant α-tocopherol and little phytosterols. This study tested whether α-tocopherol confers hepatoprotective properties while phytosterols confer hepatotoxicity to intravenous fat emulsions. Utilizing emulsions formulated in the laboratory, a soybean oil emulsion (SO) failed to protect from hepatosteatosis in mice administered a PN solution enterally. An emulsion of soybean oil containing α-tocopherol (SO+AT) preserved normal hepatic architecture. A fish oil emulsion (FO) and an emulsion of fish oil containing phytosterols (FO+P) protected from steatosis in this model. Expression of hepatic acetyl CoA carboxylase (ACC) and peroxisome proliferator-activated receptor gamma (PPARγ), was increased in animals administered SO. ACC and PPARγ levels were comparable to chow-fed controls in animals receiving SO+AT, FO, and FO+P. This study suggests a hepatoprotective role for α-tocopherol in liver injury induced by the enteral administration of a parenteral nutrition solution. Phytosterols do not appear to compromise the hepatoprotective effects of fish oil.

## Introduction

Parenteral nutrition (PN) is the intravenous administration of macronutrients and micronutrients, including carbohydrates, protein in the form of amino acids, lipids, vitamins, and trace elements. PN is a critical component of therapy for patients with intestinal failure (IF) who are unable to absorb sufficient nutrients ingested orally due to inadequate intestinal length or intestinal malfunction. Although PN is life sustaining for IF patients, there are complications associated with its administration. One such complication is the development of intestinal failure-associated liver disease (IFALD), which is characterized by cholestatic liver disease that can progress to cirrhosis and end-stage liver disease necessitating liver transplantation. Traditionally, the progression of IFALD could only be stopped if patients could wean off PN and achieve enteral autonomy. More recently, it has been demonstrated that use of fish oil as a parenteral fat source can prevent PN-induced liver injury in animal models [1,2] and reverse cholestasis and stop or slow the progression of liver disease in patients with IFALD [3–9].

Fat is an important component of PN. Fat in PN is an energy-dense calorie source as well as a source of the long-chain polyunsaturated essential fatty acids (EFA), which include those from the omega-3 and omega-6 fatty acid families. Administration of fat-free PN requires excess carbohydrate calories to meet caloric demand. Provision of fat-free PN also results in the development of essential fatty acid deficiency (EFAD), which may be characterized by dermatitis, hair loss, developmental delay, and growth impairment [10,11]. PN-dependent patients can be biochemically monitored for EFAD through serum fatty acid profiling and measurement of the ratio of the nonessential omega-9 fatty acid mead acid, which is a triene, to the essential omega-6 fatty acid arachidonic acid, which is a tetraene. The biochemical definition of EFAD is a triene to tetraene ratio greater than 0.2 [12].

Fat in PN is administered as an oil-in-water emulsion in which the oil is dispersed as globules surrounded by a phospholipid monolayer within an aqueous medium. Globules must be small enough to travel in the circulation without causing embolic events. In the United States, the United States Pharmacopeia (USP) has set standards that intravenous fat emulsions must have a mean globule size of less than 500nm in diameter and a percentage of fat globules greater than 5μm in diameter (PFAT5) of no more than 0.05% [13,14]. The types and proportions of fatty acids administered are determined by the composition of the oils used to formulate the emulsion. Oils may also contain naturally occurring non-triglyceride components or additives that are incorporated into emulsions formulated with such oils.

Soybean oil-based fat emulsions (SO) are the most commonly used parenteral fat sources. Until recently in the United States, the only parenteral fat sources approved by the Food and Drug Administration (FDA) contained soybean oil. Exposure to SO can exacerbate the risk of developing PNALD [15]. Conversely, intravenous fish oil emulsions (FO) have been shown to prevent (1, 2) PN-induced liver injury in animal models. When administered as the sole parenteral fat source to patients who develop IFALD, FO can reverse cholestasis and stop the progression of liver disease (3–9). While the mechanisms for the hepatoprotective properties of FO and hepatotoxic properties of SO are not completely understood, there are several differences between fish oil and soybean oil that may be important contributors to the differential effects of SO and FO on the liver.

Soybean oil is naturally abundant in phytosterols, which are plant-based sterol compounds. The commercially available SO contains approximately 450mg/L of phytosterols [16]. The predominant phytosterol in the commercial SO is beta-sitosterol, comprising ~70% of the total phytosterols [16]. Stigmasterol and campesterol are present in smaller, but significant quantities, ~15% and ~13% respectively [16]. *In vitro* studies have demonstrated that stigmasterol can inhibit the expression of the bile acid transporter Farsenoid X Receptor (FXR) as well as genes modulated by FXR [17]. In a murine model of IFALD, stigmasterol could exacerbate liver injury, suppress activation of bile acid transporters, and cause hepatic macrophage activation [18]. In contrast to SO, FO contains only trace amounts of phytosterols, which could represent one mechanism for the hepatoprotective properties of FO.

Alpha-tocopherol is an anti-oxidant that is an important additive in fish oil to prevent oxidation of the long-chain polyunsaturated omega-3 fatty acids. Soybean oil contains fewer omega-3 fatty acids than fish oil and does not require the addition of α-tocopherol to maintain stability. It has been shown that α-tocopherol has anti-inflammatory and anti-oxidant properties [19,20]. In a preterm piglet model of IFALD, α-tocopherol added to commercial SO improved bile acid clearance that was impaired with the administration of SO alone [21].

The purpose of this study is to test the hypothesis that α-tocopherol contributes hepatoprotective properties and phytosterols contribute hepatotoxic properties to intravenous fat emulsions. In order to test this hypothesis, it is not possible to utilize commercially available intravenous fat emulsions, as SO and FO with varying levels of phytosterols and α-tocopherol do not exist. Therefore, we formulated emulsions in the laboratory to allow for both control of the amount of α-tocopherol and phytosterols in each emulsion and to ensure uniformity of all emulsion components with variation in only the oil type. We have previously demonstrated that SO and FO formulated in the laboratory are safe and well tolerated in mice [22]. We have also found that the FO and SO made in the laboratory have the same effects on the liver as their commercial counterparts in a murine model of PN-induced liver injury (S1 Fig). Here, we tested whether the addition of phytosterols to fish oil rendered FO hepatotoxic, and whether the addition of α-tocopherol to soybean oil rendered SO hepatoprotective, with the aim of developing an understanding of the mechanisms by which FO protects the liver from PN-induced injury.

## Materials and methods

### Lipid emulsion formulation

#### Materials for emulsions

Sterile water for injection (SWFI, Hospira, Lake Forest, IL), Egg phospholipid (Lipoid LLC, Newark, NJ), Sodium Oleate (Lipoid LLC, Newark NJ), and Glycerin (Sigma-Aldrich, St. Louis, MO) were used to formulate the dispersion. Oils used were USP-grade soybean oil (Spectrum Chemicals, New Brunswick, NJ) and CrystalPure EPA 28/12 TG fish oil (Pronova Biopharma, Oslo, Norway). Additives used were α-tocopherol (Sigma-Aldrich, St. Louis, MO), beta-sitosterol (Sigma-Aldrich, St. Louis, MO), and stigmasterol (Sigma-Aldrich, St. Louis, MO). Commercial emulsions used for analyses included Omegaven (Fresenius Kabi, Bad Homburg, Germany) and Intralipid (Fresenius Kabi, Uppsala, Sweden).

#### Preparation of FO+P oil

CrystalPure EPA 28/12 TG fish oil was heated to maintain temperature between 50–60°C under constant stirring conditions. Phytosterols were added (85% beta-sitosterol, 15% stigmasterol) to a final concentration of 2.25mg phytosterols per gram of oil and stirred until dissolved. When used to formulate a 20% emulsion, the calculated phytosterol concentration was 450mg phytosterols per liter emulsion.

#### Preparation of SO+AT oil

Soybean oil was heated to maintain temperature between 50–60°C under constant stirring conditions. Alpha-tocopherol was added to a final concentration of 1mg α-tocopherol per gram of oil and stirred for 10–15 minutes. When used to formulate a 20% emulsion, the calculated α–tocopherol content was 200mg α–tocopherol per liter emulsion.

#### Emulsion formulation

Emulsions were formulated via high-pressure homogenization as previously described [22]. All steps were performed at 40–45°C unless otherwise specified and under a nitrogen atmosphere.

A dispersion was first formulated by adding frozen egg phospholipid to SWFI heated to 75–90°C under high-speed shear mixing conditions and allowing the mixture to equilibrate at 40–45°C. Sodium oleate was added and shear mixing continued (4000–4100 RPM) for 40 minutes, after which glycerin was added. The crude dispersion was homogenized (Panda Plus Homogenizer, GEA Niro Saovi, Columbia, MD) at 9000psi for 20 cycles. The dispersion was filtered through a 0.45um membrane and pH adjusted to 10.4 with 0.5N sodium hydroxide. The final dispersion was composed of 12% egg phospholipid, 25% glycerin, and 0.3% sodium oleate. One batch of dispersion was sufficient for the formulation of five 1-liter emulsions.

Emulsions were formulated by adding oil to an appropriate volume of dispersion under high-speed shear mixing conditions (3800–4200 RPM, adjusted to avoid foaming), with mixing continued for 40–45 minutes and slowly brought to a final volume of 500mL with SWFI, maintaining the temperature at 40–45°C. The crude emulsion was homogenized at 5000psi for at least 9 cycles. The final emulsion was pH adjusted to 9–9.5 using 0.1N sodium hydroxide, packaged in 20mL serum vials with head spaces flooded with nitrogen gas, and the packaged emulsions autoclaved. Final emulsion composition was 20% oil, 1.2% egg phospholipid, 2.5% glycerin, and 0.03% sodium oleate.

All emulsions underwent mean globule size and PFAT5 testing (Micro Measurement Laboratories, Deerfield, IL) in accordance with USP <729> standards [13, 14]). This includes Method I, which tests mean globule size by a light scattering technique; and Method II which determines the PFAT5 by single particle optical sizing.

#### Determination of phytosterol and alpha-tocopherol levels in emulsions

To determine phytosterol levels, samples were saponified with 2mol/L ethanolic KOH and sterols extracted with n-Heptane. Extracts were evaporated and separated on a capillary gas chromatography column. Detection was with flame ionization detector. Quantification was performed using epicoprostanol as an internal control. Alpha-tocopherol levels were determined as described [23], however instead of using an internal calibration, external calibration was used.

### Murine model of PN-induced liver injury

All animal experiments were approved by the Boston Children’s Hospital Institutional Animal Care and Use Committee. Six week-old C57BL/6 mice (Jackson Labs, Bar Harbor, ME) were administered either a standard chow diet or an enteral liquid diet composed of the PN administered to patients at Boston Children’s Hospital (20% Dextrose, 2% amino acids, 30mEq/L sodium, 20mEq/L potassium, 15mEq/L calcium, 10mEq/L magnesium, 10mMol/L phosphate, 36.67mEq/L chloride, 19.4mEq/L acetate, pediatric multivitamins, pediatric trace elements). Animals were permitted ad lib feeding of their respective diets. This model, in which mice are enterally fed a diet consisting of parenteral nutrition solution, reproducible results in liver injury in the form of hepatosteatosis over 19 days. The effect of intravenous lipids in this model has recapitulated the effects of intravenous lipids in patients with IFALD [1, 2, 4, 5, 7]. PN-fed mice were divided into groups. Each group of PN-fed mice receiving one type of fat source: IV FO, IV FO+P, IV SO, or IV SO+AT (2.4g/kg/day by tail vein injection). One additional PN-fed group served as a control and was administered intravenous (IV) saline (PN+S) at the time the other PN-fed groups were being administered an intravenous fat. There was additionally a chow-fed control group. After 19 days, animals were euthanized by carbon dioxide asphyxiation. Blood was drawn for serum collection. Livers, spleens, and the right kidney were procured for further analysis. Ten mice per treatment group were used. For the experiment shown in Supplemental Figure 1, the same murine model was utilized, however the IV lipid emulsions utilized were SO and FO (soybean and fish oil emulsions formulated in the laboratory), the commercial soybean oil emulsions Intralipid (IL, Fresenius Kabi, Uppsala, Norway) and the commercial fish oil emulsion Omegaven (OM, Fresenius Kabi, Bad Homburg, Germany).

### Organ processing and histology

Spleens, kidneys, and one portion of each liver were placed in 10% formalin and stored at 4°C for 24 hours, then transferred to 70% ethanol. Samples were embedded in paraffin and sectioned for Hematoxylin and Eosin (H&E) staining to assess hepatic architecture. A second portion of each liver was placed in Optimum Cutting Temperature (OCT) medium (Fisher Scientific, Pittsburgh, PA) and frozen in liquid nitrogen. Samples underwent frozen sectioning and oil red O staining to assess hepatic fat accumulation. Visualization was with a Zeiss Axiophot microscope (Oberkochen, Germany). Slides were analyzed by a board-certified pathologist who was blinded to the treatment groups. A third portion of each liver was flash-frozen in liquid nitrogen and stored at -80°C for gene and protein expression analysis.

### Serum fatty acid profiling

Serum fatty acid extraction was performed as previously described [2]. Briefly, serum samples (30μL per sample) with tricosanoic acid added as an internal standard underwent chloroform and methanol extraction in a ratio of 2:1 to isolate the lipid fraction. Samples were saponified with 0.5N methanolic sodium hydroxide. Samples were incubated in 14% BF_3_/methanol for 30 minutes at 100°C. Steps were performed under nitrogen gas atmosphere to minimize oxidation. Analysis was performed with gas liquid chromatography (Agilent Technologies 6890N) coupled to an Agilent-5975B mass spectrometer equipped with a Supelcowax SP-10 capillary column. An external fatty acid methyl ester standard (NuCheck Prep, Elysian, MN) was used to identify sample fatty acid peaks.

### Gene expression analysis

Livers were cut to 25mg per sample and RNA was extracted using the Qiagen AllPrep DNA/RNA/Protein kit (Gaithersburg, MD) according to the manufacturer’s instructions. For each reaction, Taqman primers (Invitrogen, Carlsbad, CA) and reagents (Agilent Technologies, Santa Clara, CA) were used according to the manufacturer’s instructions with 200ng RNA. A 2-step cycling RT-PCR protocol was used in an ABI One Step Plus cycler. An initial reverse transcription step of 30 minutes at 50°C and 10 minutes at 95°C was followed by an amplification step consisting 15 seconds at 95°C and 1 minute at 60°C cycled 40 times. Target gene expression was normalized to the GAPDH gene and compared to the chow-fed control group using the 2^-ΔΔCt^ method [24].

### Protein analysis

Livers were cut to 25mg per sample and homogenized in radioimmunoprecipitation assay (RIPA) buffer with protease inhibitor and phosphatase inhibitor using stainless steel beads in a Bullet Blender. Protein concentrations were determined using a Bradford Assay (Bio-Rad, Hercules, CA). Ten milligrams of protein per sample was separated using a 4–12% Bis Tris polyacrylamide gel (Invitrogen, Carlsbad, CA) before being transferred to a nitrocellulose membrane. Membranes were blocked in 5% non-fat milk for 1 hour. Membranes were incubated in primary antibody overnight and in secondary antibody for 1 hour. ACC and PPARγ antibodies were from Cell Signaling technologies (Danvers, MA). Beta-actin antibody was from Santa Cruz Biotechnologies (Paso Robles, CA).

### Statistical analysis

Statistics were performed with the GraphPad Prism data analysis software (La Jolla, CA). Linear regression analysis was used to compare the growth curves of the treatment groups. Single-factor analysis of variance with Tukey’s multiple comparison test was used for analysis of RT-PCR, Western blot quantification, fatty acid analysis, and body/organ mass data.

## Results

### Emulsion analysis

The following oils were used to make 20% oil in water emulsions: fish oil, fish oil to which phytosterols had been added (FO+P), soybean oil, and soybean oil to which α-tocopherol had been added (SO+AT). Table 1 shows the phytosterol and α-tocopherol levels in the emulsions made with these oils, as well as in commercially available FO (Omegaven, Fresenius-Kabi, Bad Homburg, Germany) and SO (Intralipid, Baxter, Uppsala, Sweden). Phytosterol levels in the emulsions formulated with SO, SO+AT, and FO+P were comparable. Alpha-tocopherol levels in the emulsions formulated with FO, FO+P, and SO+AT were comparable. Mean globule size and PFAT5 analysis for all emulsions met USP standards (Table 2).

**Table 1:** Phytosterol and Alpha-tocopherol Levels in emulsions.

Emulsion	Phytosterol (mg/L)	Alpha-Tocopherol (mg/L)
**OM**	10	193
**IL**	570	12
**FO**	46	133
**FO+P**	424	129
**SO**	461	7
**SO+AT**	446	164

Abbreviations: OM = commercial 10% fish oil emulsion Omegaven; IL = commercial 20% soybean oil emulsion Intralipid; FO = 20% fish oil emulsion formulated in the laboratory; FO+P = 20% emulsion of fish oil with phytosterols added formulated in the laboratory; SO = 20% soybean oil emulsion formulated in the laboratory; SO+AT = 20% emulsion of soybean oil with alpha-tocopherol added formulated in the laboratory

**Table 2:** Emulsion USP <729> particle size analysis.

Emulsion	Mean Globule Size (nm)	PFAT5 (%)
**FO**	238.7 ±4.0	0.032 ±0.003
**FO+P**	242.3 ±4.7	0.015 ±0.001
**SO**	252.8 ±0.4	0.009 ±0.001
**SO+AT**	252.8 ±0.3	0.013 ±0.001

Mean globule size and percent of particles larger than 0.05nm in diameter (PFAT5). USP standards for intravenous fat emulsions are mean globule size < 500nm and PFAT5 ≤ 0.05%.

### Growth parameters and fatty acid profiles

**Fig 1:**
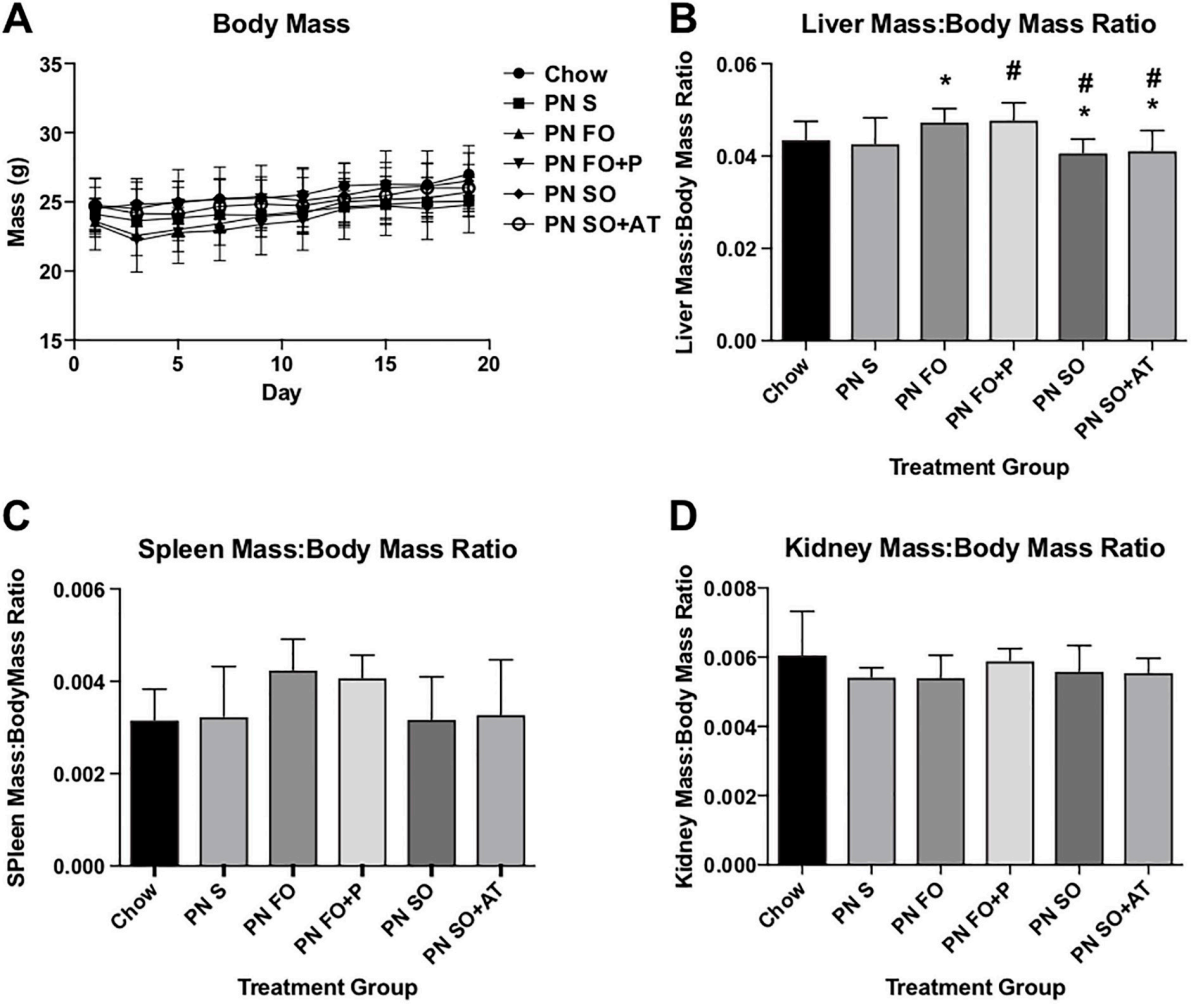
Growth parameters between groups administered the PN diet and administration of fat emulsions formulated in the laboratory. Fig 1A: Body masses were monitored every other day over the course of the enteral PN regimen. There were no differences in growth curves on linear regression analysis. Liver (Fig 1B), spleen (Fig 1C), and right kidney (Fig 1D) masses at euthanasia after 19 days of the PN diet. * = p<0.05 compared to PN FO group and PN FO+P group. N = 10 mice per treatment group. Statistical analysis of 1B, 1C, and 1D was performed with one-way ANOVA.

There were no adverse clinical effects with administration of any of the emulsions used, and animals tolerated all emulsions well. Growth curves among the treatment groups are similar on linear regression analysis (Fig 1A).

Our laboratory has previously demonstrated that intravenous infusion of unstable lipid emulsions results in enlargement of the spleen and liver, as well as histologic evidence of fat globule deposition in these organs [25]. Liver (Fig 1B), spleen (Fig 1C), and kidney (Fig 1D) masses to body mass ratio were similar in emulsion-treated groups compared to chow-fed control animals. Liver mass to body mass ratios were slightly higher in groups treated with fish oil-based emulsions compared to soybean oil-based emulsions (Fig 1B).

**Fig 2:**
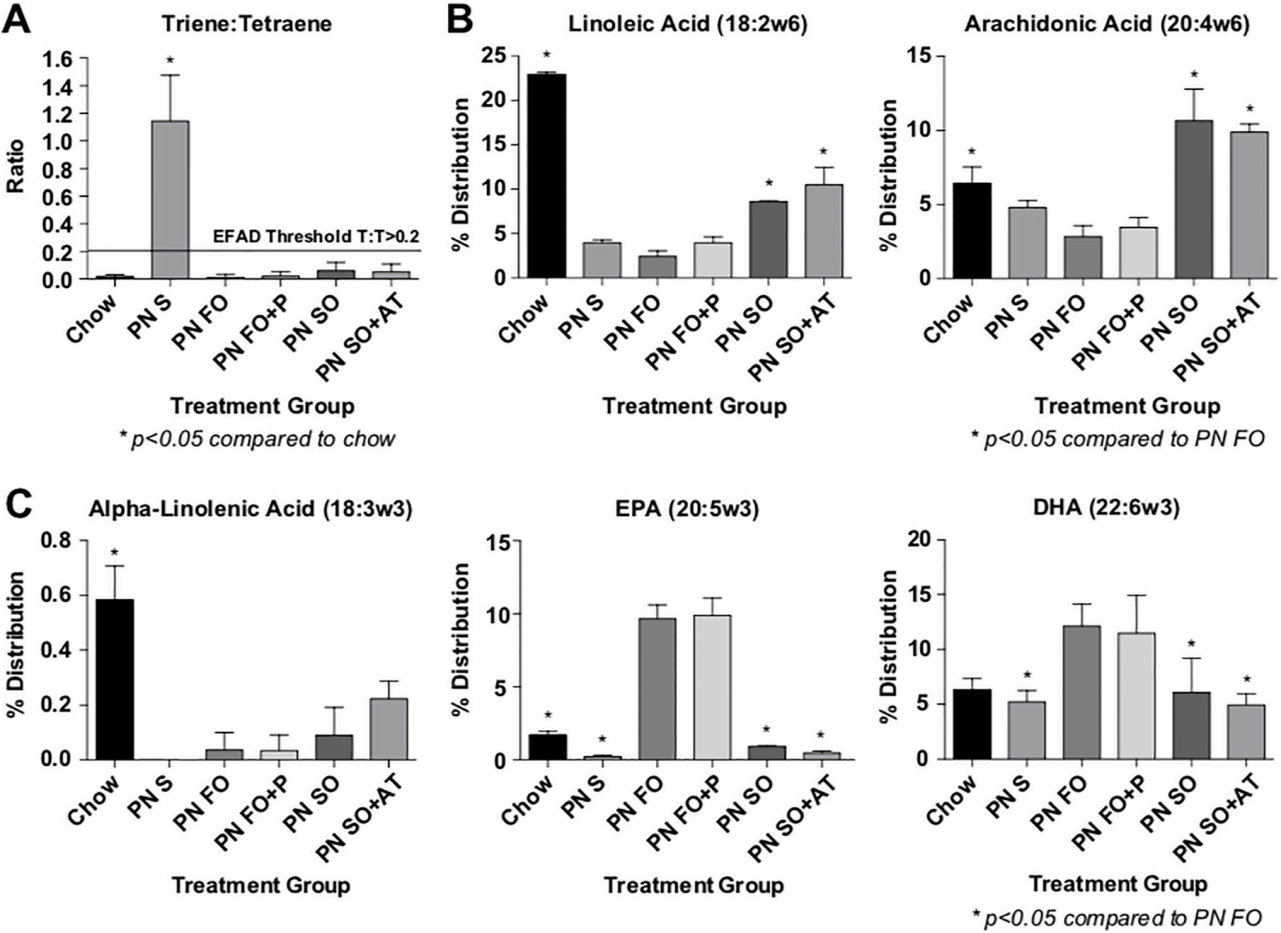
Serum fatty acid profiles after 19 days of the PN diet with administration of fat emulsions formulated in the laboratory. Fig 2A: Triene to tetraene ratios. Only mice receiving no fat source (PN+saline) met biochemical criteria for EFAD. Distribution of the main omega-6 (Fig 2B) and omega-3 fatty acids (Fig 2C) reflect the fat source administered to each group. N = 3 samples per treatment group randomly selected. Statistical analysis was performed with one-way ANOVA. * = p<0.05 with respect to the PN FO group.

Serum fatty acid profiles were performed in order to confirm that each emulsion was able to prevent EFAD and delivered the expected complement of EFA for the oil used in the emulsion. SO is abundant in omega-6 fatty acids and contains few omega-3 fatty acids, while FO is more abundant in omega-3 fatty acids and contains a paucity of omega-6 fatty acids. These EFA balances should be reflected in the serum of animals in each respective treatment group. All emulsions prevented biochemical EFAD (Fig 2A). FO and FO+P emulsions resulted in lower serum levels of the omega-6 fatty acid arachidonic acid (ARA) and higher serum levels of the omega-3 fatty acids eicosapentaenoic acid (EPA) and docosahexaenoic acid (DHA) compared to the SO and SO+AT emulsions (Fig 2B and 2C).

### Histologic analysis

**Fig 3:**
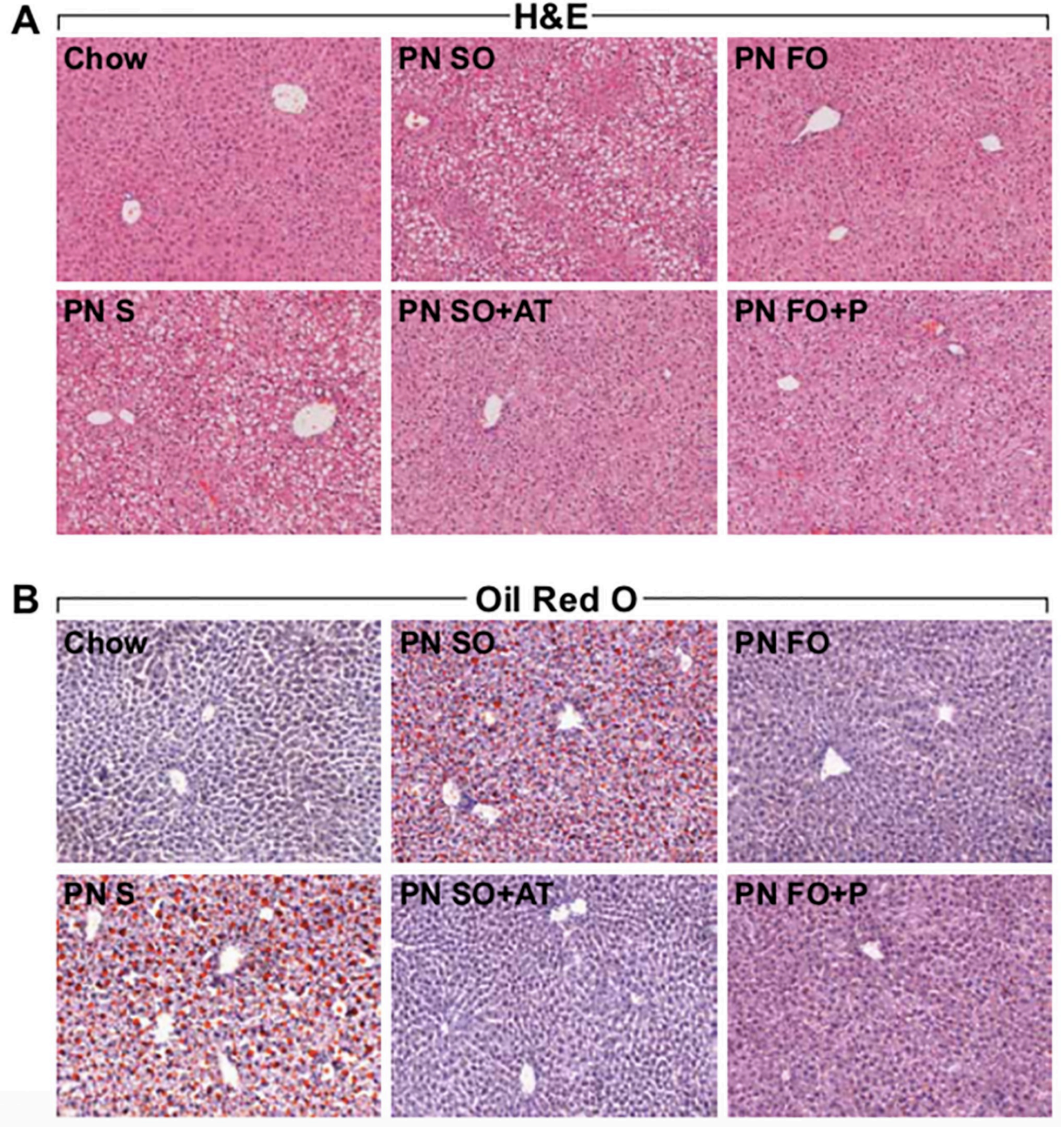
Normalization of hepatic architecture and hepatic fat accumulation with addition of α-tocopherol to SO in an intravenous fat source with the enteral PN diet. Representative Hematoxylin and Eosin (H&E, Fig 3A) and Oil Red O (Fig 3B) images demonstrating hepatic architecture and hepatic fat accumulation, respectively. N = 10 samples per treatment group for H&E (Fig 3A), and n = 3 representative samples for Oil Red O (Fig 3B). Images are 100X magnification (10X objective, 10X eyepiece).

To assess the effect of each emulsion on the development of enteral PN-induced steatosis, hepatic histologic analysis was performed. SO did not prevent steatosis (Fig 3A). However, addition of α-tocopherol to SO (SO+AT) resulted in preservation of normal hepatic architecture in PN-fed animals (Fig 3A). FO and FO+P also preserved normal hepatic architecture (Fig 3A) suggesting that the addition of phytosterols to FO does not compromise the ability of FO to protect the liver from enteral PN-induced steatosis. On Oil Red O analysis to assess hepatic fat accumulation, SO+AT resulted in decreased hepatic fat accumulation compared to SO (Fig 3B), suggesting that α-tocopherol confers hepatoprotective properties to SO. Both FO and FO+P had minimal hepatic fat accumulation, again suggesting that the addition of phytosterols to FO does not compromise the hepatoprotective properties of FO (Fig 3B).

### Molecular assessment

**Fig 4:**
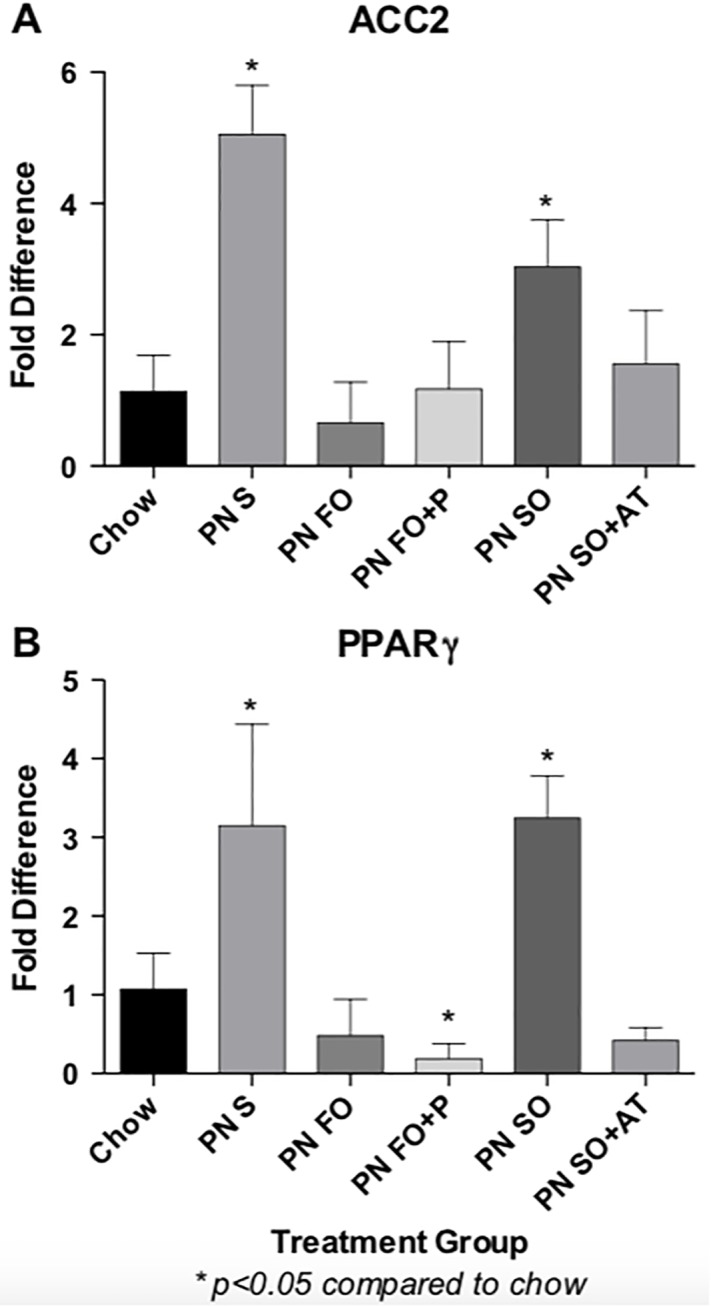
Hepatic gene expression of ACC2 and PPARg are dysregulated in the presence of enteral PN-induced liver injury. Expression of ACC2 (Fig 4A) and PPARγ (Fig 4B) is dysregulated by the fat-free PN diet and the PN diet with IV SO as a fat source, and normalized by IV FO and by the addition of α-tocopherol to SO as fat sources. Gene expression is measured as fold-difference compared to the chow-fed group. N = 5 samples per treatment group, each performed in technical duplicate. Statistical analysis was performed with one-way ANOVA.

**Fig 5:**
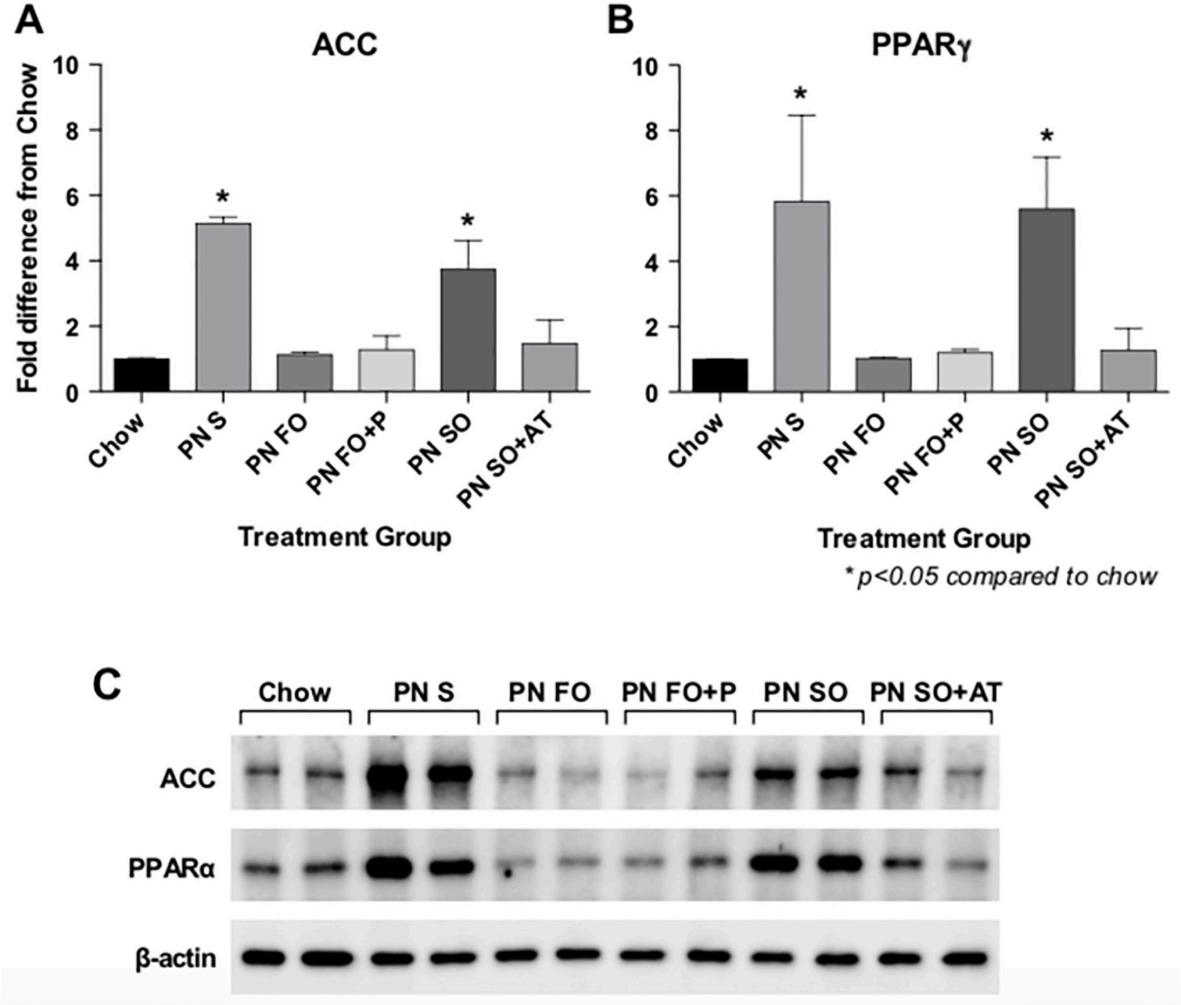
Hepatic protein expression of ACC2 and PPARg are dysregulated in the presence of enteral PN-induced liver injury. Protein levels of ACC2 (Fig 5A) and PPARγ (Fig 5B) are upregulated by the fat-free PN diet and the PN diet with IV SO as a fat source, and normalized by IV FO and by the addition of α-tocopherol to SO as fat sources. Protein levels were quantified comparatively by normalizing each group to the corresponding beta-actin level and comparing to the chow-fed group. Western Blots performed in biological duplicate for each group. Statistical analysis was performed with one-way ANOVA. Fig 5C: Western Blot image.

PN-fed mice administered IV SO developed steatosis while those on a PN diet with FO did not. Therefore, we hypothesized that SO and FO may differentially affect hepatic fat metabolism. In order to test the effect of each emulsion on hepatic fat metabolism, expression analysis of genes that regulate hepatic fat metabolism was performed. Acetyl CoA Carboxylase 2 (ACC2), which catalyzes the rate-limiting step in *de novo* lipogenesis, and Peroxisome Proliferator Activated Receptor-gamma (PPARγ), which is a transcriptional regulator of hepatic fat metabolism were identified as genes whose expression is increased with the enteral PN diet and no fat source, is normalized by the provision of IV FO, but not by provision of IV SO (Fig 4A and 4B). IV SO+AT demonstrated normalization of ACC2 and PPARγ at the gene expression (Fig 4A and 4B) and protein expression (Fig 5) levels, suggesting that the addition of α-tocopherol can impart hepatoprotective properties to SO. Both FO and FO+P resulted in normalized gene (Fig 4A and 4B) and protein (Fig 5) expression of ACC2 and PPARγ, suggesting that at the molecular level, the addition of phytosterols does not compromise the hepatoprotective properties of FO.

**Fig 6:**
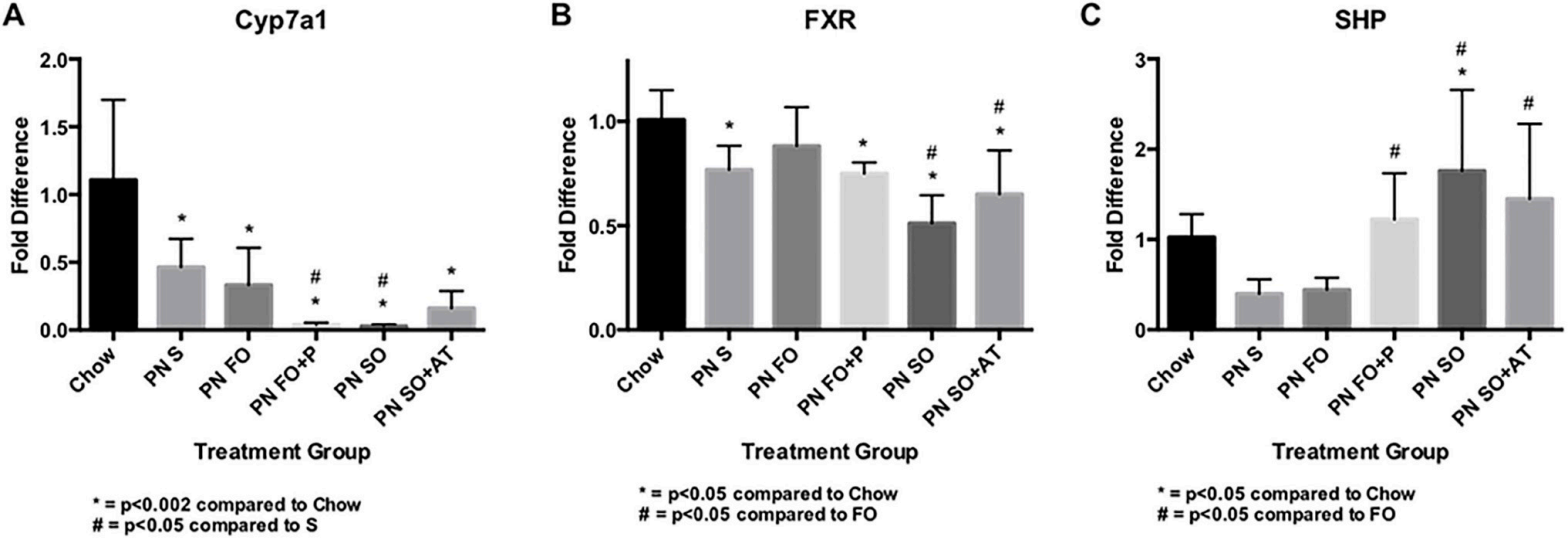
Expression of regulators of bile homeostasis with expression altered by phytosterol-containing intravenous lipids, and at least partially normalized by α-tocopherol. Data are expressed as fold difference in expression compared to the chow-fed control group. A) Cyp7a1 = regulator of bile acid synthesis; B) FXR = farsenoid X receptor; C) SHP = small heterodimer protein. N = 5 samples per group each performed in technical duplicate, and analysis was performed with single-factor ANOVA.

Histologically, our murine model demonstrates steatosis in response to the PN+SO diet. However the clinical correlate, IFALD, is characterized by cholestasis. Therefore we hypothesized that phytosterol-containing intravenous fat emulsions may cause alterations in the expression of regulators of bile synthesis and transport, and that α-tocopherol-containing intravenous fat emulsions may mitigate some of these changes. Expression of important regulators of bile acid homeostasis that were previously assayed in a neonatal piglet model of IFALD [26] was measured in this model. Cyp7a1, a regulator of bile acid synthesis, as well as the regulators of bile acid transport FXR and small heterodimer protein (SHP) demonstrated altered expression with administration of phytosterol-containing intravenous lipids that was at least partially normalized in the setting of IV FO or SO+AT administration (Fig 6). Cyp7a1 expression (Fig 6A) decreased with the PN diet. Its expression further decreased with enteral PN and intravenous phytosterol-containing lipids. Alpha-tocopherol addition to SO appeared to partially normalize its expression. FXR expression (Fig 6B) was decreased in the enteral PN groups, with SO demonstrating even lower expression compared to FO. Although there was some normalization of FXR expression in the SO_AT group, this did not reach statistical significance. SHP expression (Fig 6C) was increased in PN fed animals receiving phytosterol-containing lipids. Other regulators of bile acid transport were assayed without particular patterns of expression with respect to phytosterol and α-tocopherol (S2 Fig). Overall there was little correlation between expression of these regulators of bile acid synthesis and histologic findings.

## Discussion

The role of phytosterols and α-tocopherol in modulating the effects of intravenous fat emulsions on the liver has been debated. Serum and hepatic phytosterol levels are higher in PN-dependent patients receiving soybean oil-containing intravenous lipid emulsions than in patients who have been weaned off PN [26]. Furthermore, among PN-dependent patients, levels of serum and liver phytosterols positively correlate with the degree of deranged liver enzymes as well as the degree of portal inflammation and hepatic fibrosis on histologic analysis [26]. In neonatal PN-dependent patients, serum phytosterol levels are higher in patients who meet biochemical criteria for IFALD than in those without IFALD [27]. *In vitro* studies have shown that stigmasterol, one of the principal phytosterols in SO [16], inhibits the expression of target genes of the bile acid-responsive nuclear receptor FXR [17]. In contrast, Ng et al found no adverse effects on bile acid clearance with the addition of beta-sitosterol and stigmasterol to commercial FO in a preterm piglet model of IFALD [21]. This group did show that SO-mediated deficits in bile acid clearance were ameliorated by adding α-tocopherol to a commercial SO [21]. However, similar studies performed by Muto et al in a neonatal piglet model of IFALD showed no improvements in bile flow, serum bile acid concentration, or serum direct bilirubin levels with the addition of α-tocopherol to a commercially available SO emulsion [28].

In this study, an intravenous lipid emulsion formulated in the laboratory using soybean oil to which α-tocopherol had been added was able to preserve normal hepatic architecture and normal expression of 2 important regulators of hepatic fat metabolism in a murine model of PN-induced liver injury in which mice are enterally fed a PN solution. An intravenous lipid emulsion formulated in the laboratory using SO that did not contain added α-tocopherol was not able to protect from PN-induced hepatosteatosis and dysregulation of hepatic fat metabolism. These results are consistent with the findings of Ng et al; albeit in a different model of PN-induced liver injury utilizing intravenous lipid emulsions formulated in the laboratory. The conflicting findings of Muto et al, that did not find improvement in markers of cholestasis with the addition of α-tocopherol to a commercially available SO may have been due to a number of issues related to integration of α-tocopherol into SO as there was no FO control group to which to compare. Another explanation is small sample size, as their study utilized only 8 piglets per experimental group. Formulation of the intravenous lipid emulsions in the laboratory allowed for control of the formulation protocol, and use of the same instruments and ingredients to ensure all emulsions used in the study were made in precisely the same way. Here, α-tocopherol was added to the soybean oil prior to formulating the emulsions, which recapitulates the process for formulating commercial FO emulsions and is the way α-tocopherol is integrated into commercially available emulsions.

This study also identified PPARγ and ACC2 as genes that are dysregulated by the enteral PN diet, normalized by IV FO and α-tocopherol-containing SO, but not by SO alone. PPARγ is a transcriptional regulator of systemic and hepatic fat metabolism as well as inflammation. It has been demonstrated that the PPARγ agonist rosiglitazone can reduce hepatic inflammation and associated biomarkers in a methionine- and choline-deficient diet mouse model of nonalcoholic steatohepatitis [29]. In mice lacking the low-density lipoprotein receptor, rosiglitazone has been shown to improve high-fat diet-induced hepatosteatosis [30]. Other studies have reported a positive correlation between increased PPARγ expression and the development of hepatosteatosis and accumulation of hepatic triglycerides in murine models of nonalcoholic fatty liver disease [31–33]. The hepatic triglyceride-accumulating STAT5 knockout mice also exhibit reduced hepatic fat accumulation upon antagonism of PPARγ [34]. *In vitro* studies also suggest an adipogenic effect associated with increased expression of PPARγ [35]. Interestingly, it has also been shown that beta-sitosterol, one of the principal phytosterols in SO, upregulates expression of PPARγ in a rat model of radiation-induced oxidative stress [36]. ACC2 encodes the enzyme that catalyzes the rate-limiting step of *de novo* lipogenesis. ACC2 expression has been shown to be upregulated in response to high-fructose conditions and normalized by treatment with the omega-3 fatty acid DHA in primary murine hepatocytes [37].

Interestingly this study did not find a hepatotoxic effect of adding phytosterols to fish oil in enteral PN-induced liver injury. One possible conclusion is that the phytosterols in soybean oil are not responsible for the soybean oil-associated hepatotoxic effects in the murine model of PN-induced liver injury. An alternative explanation is that phytosterols do have hepatotoxic properties but are unable to overcome the hepatoprotective properties of fish oil. The omega-3 fatty acids abundant in fish oil are precursors of anti-inflammatory lipid mediators [38,39], and fish oil is also abundant in α-tocopherol. These properties may offer hepatoprotection that cannot be overcome by the presence of phytosterols. While results consistent with those in this study have been observed in a preterm piglet model of IFALD [21], other *in vivo* and *in vitro* studies suggest that phytosterols do have hepatotoxic properties and can affect the development of cholestasis [17,18]. This study demonstrates a role for phytosterols in intravenous lipid emulsions in altering the expression of certain regulators of bile acid homeostasis, as well as the ability of α-tocopherol to partially normalize expression of these genes. A third possible explanation is that specific phytosterols at specific concentrations, or that a certain balance of phytosterols is required for phytosterol-associated hepatotoxic properties to occur. In this study, the composition of phytosterols added to fish oil approximated the types and amounts of phytosterols found in soybean oil.

One limitation of this study is that while the manifestation of IFALD in the clinical setting is cholestasis, the murine model utilized for these studies demonstrates steatosis. Despite differences in phenotypic expression, this murine model of steatosis induced by enteral administration of a PN solution has been utilized previously; and the FO-mediated prevention of liver injury in this murine model faithfully recapitulates the effect of FO in normalizing IFALD in the clinical setting [1, 2]. Furthermore, in this study, we show that genetic regulators of bile acid homeostasis are indeed altered with the administration of phytosterol-containing intravenous lipid emulsions. It may be the case that the degree of these genetic alterations in the murine model is insufficient to result in histologic or observable functional changes.

All emulsions formulated in this study protected from the development of EFAD. Traditionally linoleic (LA) and alpha-linolenic (ALA) acids, the parent omega-6 and omega-3 fatty acids respectively, have been considered the EFAs. More recent data has suggested that provision of metabolites of LA and ALA, such as ARA, EPA, and DHA is sufficient to prevent the development of EFAD while ensuring adequate growth and reproduction function [40]. Interestingly, this study found that serum ARA, EPA, and DHA reflected the balance of EFAs provided by the emulsion administered rather than serum LA and ALA levels.

The ability of α-tocopherol to render SO less hepatotoxic in a model of enteral PN-induced liver injury implies that α-tocopherol may be useful in the clinical management of IFALD and other similar hepatic pathologies. Currently FO may be used for the treatment of IFALD, however FO is not readily available to all patients. The benefits of α-tocopherol in the management of IFALD remain to be tested clinically, and it is likely that FO has additional properties, such as an abundance of omega-3 fatty acids, that render FO more beneficial than α-tocopherol alone. However, the results of this study suggest that α-tocopherol may be an option in the prevention and/or treatment of IFALD in patients for whom FO is not available.

## Supporting information

S1 Fig Fat emulsions formulated in the laboratory recapitulate the effects of similar commercially available intravenous fat emulsions in a murine model of PN-induced liver injury. Fat-free PN results in the development of steatosis over 19 days (bottom left panel). FO formulated in the laboratory (PN+FO) and commercially available FO emulsion (OM) preserve normal hepatic architecture with the PN diet, while SO formulated in the laboratory (PN+SO) and commercially available SO (IL) do not.
(TIFF)

S2 Fig Regulators of bile acid homeostasis not significantly affected by modulation of phytosterols and α-tocopherol in intravenous lipid emulsions. Data are expressed as fold difference compared to the chow-fed control group. A) BSEP = bile salt export pump; B) NTCP = Na+-taurocholate cotransporting polypeptide; C) MRP3 = multidrug resistance protein-3; D) MRP2 = multidrug resistance protein-2. N = 5 samples per group, each performed in technical duplicate. Statistical analysis was performed using single-factor ANOVA.
(TIFF)

S3 Fig Raw data to support Fig 1. Sheet 1: Murine masses over the course of the 19-day experiment that were utilized to generate Fig 1A. Sheet 2: Murine masses and organ masses on Day 19 of the experiment at euthanasia that were utilized to generate Fig 1B.
(XLSX)

S4 Fig Raw data to support Fig 2. Each sheet filled in contains the data for a single sample (as named in the sheet title). Areas under the peak curves supplied by the core facility running the samples were entered and normalized to the response for fatty acid 17:0 (Column D). These values were used to calculate the mass of each fatty acid within the sample (Column G) and the percent distribution of each fatty acid (Column L). The percent distribution of fatty acids is which is shown in Fig 2B and 2C. Triene to tetraene ratio (Fig 2A) was calculated by dividing the mass of mead acid (20:3w9) to arachidonic acid (20:4w6), this calculation appears in cell C41 of each sheet.
(XLS)

S5 Fig Raw data to support Fig 4. Sheet 1 shows the calculations used to generate Fig 4A. Sheet 2 shows the calculations used to generate Fig 4B.
(XLSX)

S6 Fig Raw Data to support Fig 5A and 5B. Columns B-E represent the calculations used to generate Fig 5B. Columns F-I represent the calculations used to generate Fig 5A. Columns J and K are the values for beta-actin.
(XLSX)

S7 Fig Raw data to support Fig 5C. Full image of the gel stained for ACC.
(TIF)

S8 Fig Raw data to support Fig 5C. Full image of gel stained for PPARγ.
(TIF)

S9 Fig Raw data to support Fig 5C. Full image of gel stained for beta-actin.
(TIF)

S10 Fig Raw data to support Fig 6. Sheet 1 shows the data and calculations used to generate Fig 6A. Sheet 2 shows the data and calculations utilized to generate Fig 6B. Sheet 3 shows the data and calculations utilized to generate Fig 6C.
(XLSX)

S11 Fig Raw data to support S2 Fig. Sheet 1 shows the data and calculations utilized to generate S2A Fig. Sheet 2 shows the data and calculations utilized to generate S2B Fig. Sheet 3 shows the data and calculations utilized to generate S2C Fig. Sheet 4 shows the data and calculations utilized to generate S2D Fig.
(XLSX)
