# Supplementary figures and images for: Alpha-tocopherol in intravenous lipid emulsions imparts hepatic protection in a murine model of hepatosteatosis induced by the enteral administration of a parenteral nutrition solution
Source: PLoS One. 2019 Jul 11;14(7):e0217155. doi: 10.1371/journal.pone.0217155 (PMC6622470; doi:10.1371/journal.pone.0217155)

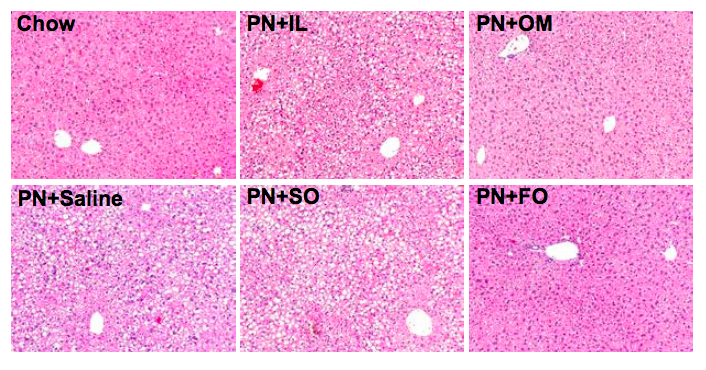

Supplement: S1 Fig — Fat-free PN results in the development of steatosis over 19 days (bottom left panel). FO formulated in the laboratory (PN+FO) and commercially available FO emulsion (OM) preserve normal hepatic architecture with the PN diet, while SO formulated in the laboratory (PN+SO) and commercially available SO (IL) do not. (TIFF) [file pone.0217155.s001.tiff]

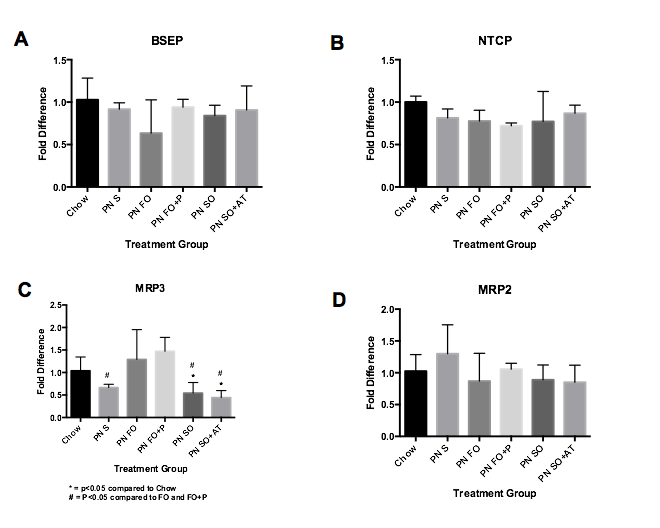

Supplement: S2 Fig — Data are expressed as fold difference compared to the chow-fed control group. A) BSEP = bile salt export pump; B) NTCP = Na+-taurocholate cotransporting polypeptide; C) MRP3 = multidrug resistance protein-3; D) MRP2 = multidrug resistance protein-2. N = 5 samples per group, each performed in technical duplicate. Statistical analysis was performed using single-factor ANOVA. (TIFF) [file pone.0217155.s002.tiff]

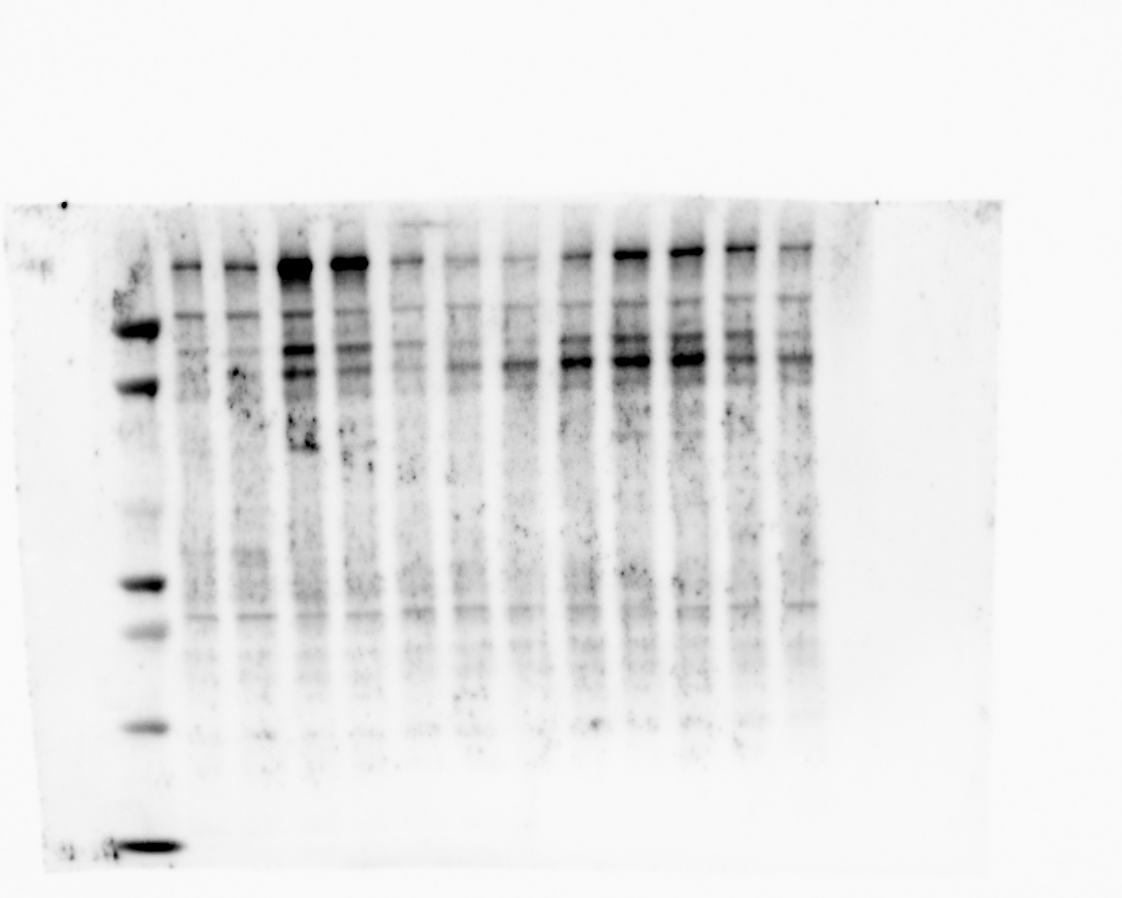

Supplement: S7 Fig — Full image of the gel stained for ACC. (TIF) [file pone.0217155.s007.tif]

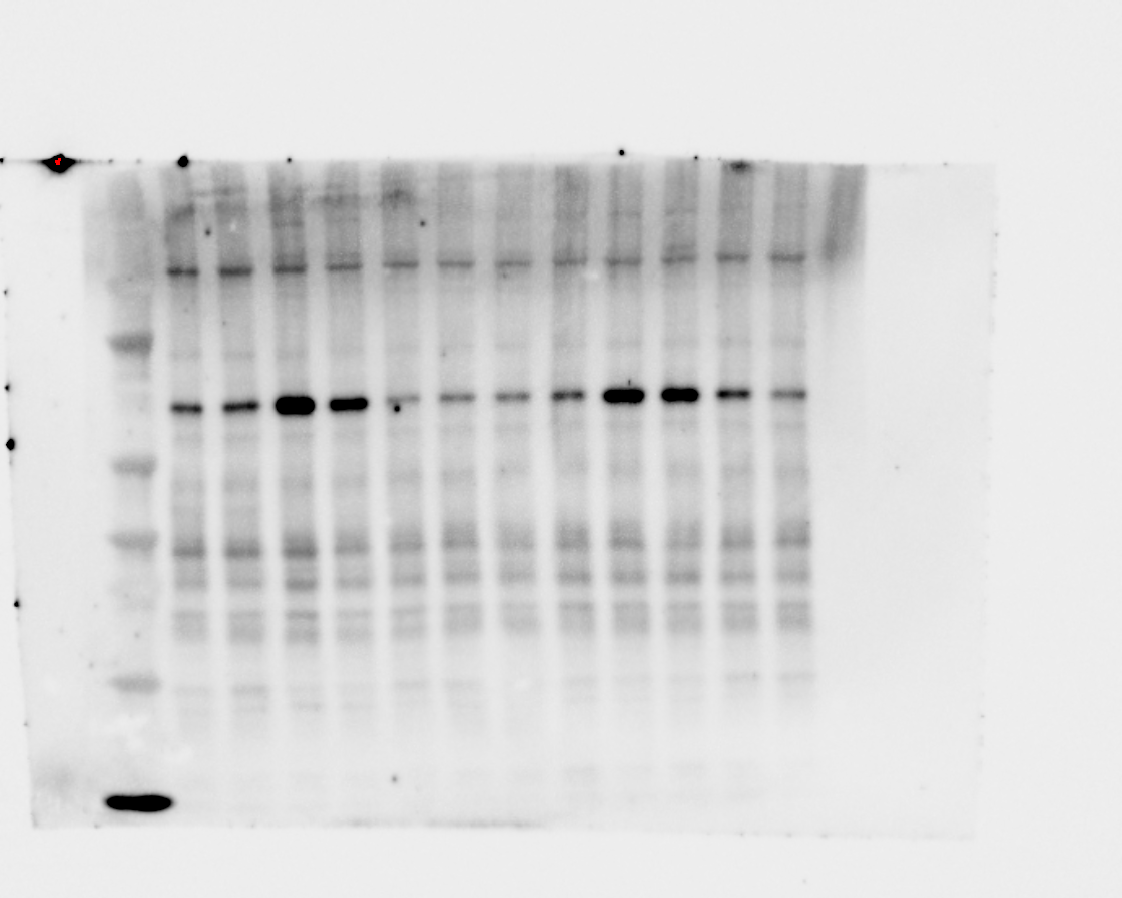

Supplement: S8 Fig — Full image of gel stained for PPARγ. (TIF) [file pone.0217155.s008.tif]

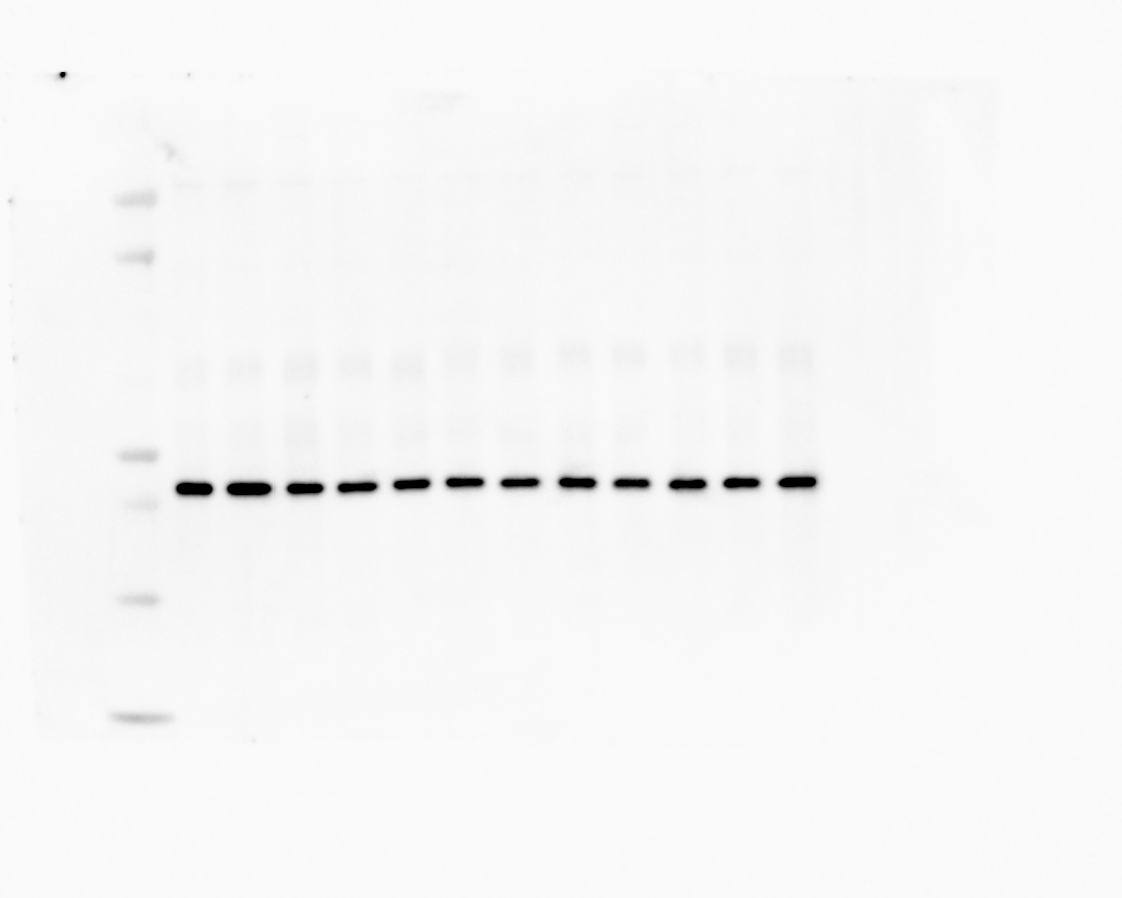

Supplement: S9 Fig — Full image of gel stained for beta-actin. (TIF) [file pone.0217155.s009.tif]
